# Moral Leadership and Unethical Pro-organizational Behavior: A Moderated Mediation Model

**DOI:** 10.3389/fpsyg.2019.02640

**Published:** 2019-11-28

**Authors:** Yujuan Wang, Hai Li

**Affiliations:** ^1^Department of Human Resource Management, Business School, Beijing Normal University, Beijing, China; ^2^Faculty of Business Administration, Shanxi University of Finance and Economics, Taiyuan, China

**Keywords:** moral leadership, identification with supervisors, taking responsibility, moral courage, unethical pro-organizational behavior

## Abstract

In this paper, we aim to examine the indirect effects of moral leadership on unethical pro-organizational behavior (UPB). Drawing on Social Identity Theory, identification with supervisors (social identity) and taking responsibility (personal identity) were hypothesized as mediators linking moral leadership and UPB. In addition, we aim to investigate the moderating role of moral courage in the relationship between moral leadership and UPB. We conducted two studies with two distinct samples: one on a sample of 161 MBA students, and the other on a sample of 205 enterprise employees in China. Data were collected through a self-reported questionnaire based on a two-wave research design and analyzed through Structural Equation Modeling. Results showed that moral leadership increased UPB through promoting identification with supervisors while reducing UPB *via* increasing taking responsibility. Additionally, the results also showed that moral courage moderated the mediating effects of identification with supervisors and taking responsibility upon the relationship between moral leadership and UPB. We contribute to the literature by demonstrating that moral leadership exerts its paradoxical effects on UPB indirectly through its impact on identification with supervisors and taking responsibility and therefore offers a better understanding of how and when moral leadership influences UPB. A number of managerial implications are also discussed.

## Introduction

With rapid economic development, while the positive change from the business benefits our lives, we also witness endless business scandals, such as the Enron Incident, the Sanlu Company Melamine Incident, and the Volkswagen diesel emissions scandal. Because of this, scholars have recently begun to pay increased attention to unethical workplace behavior (Moore and Gino, 2013; Treviño et al., 2014), much of it focused on the self-serving unethical behavior (Moore and Gino, 2013; Treviño et al., 2014). However, some employees engage in accounting fraud to protect the organization (Amernic and Craig, 2010) or bribe officials to get ahead of competitors (Effelsberg et al., 2014). In the academic literature, such behaviors are called “unethical pro-organizational behaviors” (UPBs) (Umphress et al., 2010).

In contrast to general unethical behavior, UPB has been defined as “actions that are intended to promote the effective function of the organization or its members and violate core societal values, mores, laws, or standards of proper conduct” (Umphress and Bingham, 2011, p. 622). The definition illustrates two characteristics of UPB, which refers to behavior that is unethical but benefits the organization. When UPB violates widely held moral standards, it can greatly affect not only the organization but also people outside of the organization (Vadera and Pratt, 2013), which results in ruin for the organizational reputation and harm to the interests of external stakeholders and society overall (Umphress and Bingham, 2011).

While acknowledging the potential consequences of UPB, scholars have explored the antecedents of UPB (e.g., Chen et al., 2016; Castille et al., 2018; Xu and Lv, 2018). Previous studies have examined personal factors (Machiavellianism, organizational identification, affective commitment, psychological entitlement) (cf. Umphress et al., 2010; Matherne and Litchfield, 2012; Chen et al., 2016; Castille et al., 2018; Lee et al., 2019), workplace situational factors (job insecurity; interpersonal justice, overall justice, social exclusion, perceived organizational support, positive social exchange, employee-organization relationship) (cf. Ilie, 2012; Thau et al., 2015; Ghosh, 2017; Bryant and Merritt, 2019; Wang et al., 2019), organizational factors (workplace spirituality, high performance work systems, high performance expectation, idiosyncratic deals) (cf. Ilie, 2012; Chen and Liang, 2017; Xu and Lv, 2018; Zhang, 2018), and leadership factors (ethical leadership, transformational leadership) (cf. Miao et al., 2013; Effelsberg et al., 2014; Kalshoven et al., 2016). However, leadership, as an important organizational context variable in shaping subordinates’ behaviors (Chen et al., 2002), has not been sufficiently focused on. In particular, moral leadership emphasizes personal virtues (e.g., integrity, selflessness, altruism and accountability) and role modeling (Cheng et al., 2004) beyond that of ethical leadership. Subsequently, moral leadership has an effect on employees’ identification with and trust in their leader (e.g., Wu et al., 2012; Gu et al., 2015) which, in turn, may influence employees’ behaviors. Extant research indicates that identification is an important psychological mechanism in promoting UPB (Umphress et al., 2010; Chen et al., 2016; Johnson and Umphress, 2019). Therefore, we propose that moral leadership may be one avenue that shapes employees’ identification and thus impacts their UPB.

Further, existing research on the relationship between positive leadership and UPB mainly focuses on the following two perspectives: social exchange, which states that ethical leadership increases subordinates UPB by evoking reciprocal behavior (Miao et al., 2013; Kalshoven et al., 2016), and social learning, which states that ethical leadership decreases subordinates UPB through modeling ethical behavior for subordinates to emulate (Miao et al., 2013). Although the two perspectives help us to understand the mechanism of positive leadership’s impact on UPB from different aspects, they are limited to the external guidance of leaders while neglecting the intrinsic motivation of employees engaging in or resisting UPB. Hence, it is insufficient to answer why employees are willing to risk engaging in UPB or to resist UPB consciously. To our knowledge, only two studies elaborated the relationship between positive leadership and UPB through the lens of identification (Effelsberg et al., 2014; Kalshoven et al., 2016), which examined only the dark side of positive leadership through enhancing employees’ organizational identification. However, the basic proposition of Social Identity Theory is that identification involves the incorporation of the referent person’s attributes or group’s norms into an individual’s self-concept (Tajfel and Turner, 1986), which may be classified as either personal identity or social identity (Banaji and Prentice, 1994; Brewer and Gardner, 1996). During identification, a variety of outcomes occur due to individual doing for fulfilling his/her needs (Ashforth et al., 2008; Conroy et al., 2017), including belongingness (need for interpersonal attachments and feelings of similarity to a person or a group) and self-consistency (need for thinking and behaving in ways that perpetuate self-concept). Thus, we argue that moral leadership may be an important antecedent that contains different paths and thus has different impacts on UPB. It is necessary for us to explore the psychological mechanisms underlying the process by which moral leadership impacts UPB.

Therefore, the purpose of our study is to examine whether and how moral leadership impacts UPB through shaping employees’ different identities. Drawing on Social Identity Theory, we propose two competing mediating mechanisms of moral leadership’s impact on UPB: identification with supervisors as social identity reflecting employees’ belongingness and taking responsibility as personal identity reflecting employees’ self-consistency. The core component of Social Identity Theory is the formation of the individual’s self-concept (Tajfel and Turner, 1986). Research indicates that shaping subordinates’ self-concept is a vital practice for leaders to have an impact upon their subordinates (Kark et al., 2003). Moral leaders emphasize integrity, selflessness, altruism and never taking advantage of others (Cheng et al., 2000; Farh et al., 2008), which is likely to increase the subordinates’ identification with leaders—the sense of oneness with the leaders reflects employees’ needs for belongingness. In support of belongingness, employees may be willing to risk disregarding the social moral standards to protect and support their leaders and the organizations they represent and thus engage in UPB. Meanwhile, moral leaders set an example for their subordinates by demonstrating self-discipline, fulfilling obligations, and behaving as a selfless paragon (Farh et al., 2008). This example is likely to be transmitted to employees and thus motivates employees to define themselves as responsible persons—an identity of concerning about the effects of any act they committed and taking responsibility for their actions reflects employees’ need for self-consistency. In order to support self-consistency, employees may be willing to attach importance to the social moral standards and thus reduce UPB. Therefore, moral leadership could have both positive and negative effects on UPB through two different mechanisms.

**FIGURE 1:**
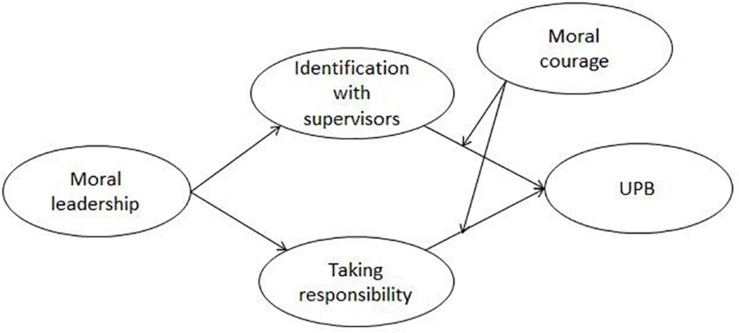
The research model.

Further, we extend our theorizing of the underlying mechanisms between moral leadership and UPB through identifying the moderating role of a personal characteristic, moral courage, which is a critical factor in predicting whether employees act in line with their moral judgments (Hannah et al., 2011). According to Social Identity Theory, the impact of identity that is activated depends not only on situational factors but also on personal characteristics (Stets and Burke, 2000). High moral courage means a high strength level of adhering to moral principles and acting ethically (Hannah et al., 2011). Thus, compared with low moral courage, high moral courage is more likely to mitigate the positive correlation between identification with supervisors and UPB, while enhancing the negative correlation between taking responsibility and UPB. Altogether, our research model is presented in Figure 1.

Our study makes several contributions to the literature on UPB, leadership, and Social Identity Theory. First, we enrich the extant research on the impact factor of UPB by examining the role of moral leadership. Although it is reasonable for the logical linkage between moral leadership and UPB by motivating employees to be responsible for their actions and to be concerned about the effects of any act, the true impact of moral leadership on UPB is still unknown. Incorporating moral leadership to our research model helps us in better understanding the antecedents of UPB. Second, we bring a comprehensive understanding about moral leadership and its effect on UPB. Extant research focuses on the positive effect of moral leadership (Farh et al., 2006), while we know little about the dark side of moral leadership. We attempt to theoretically and empirically examine the double-edged sword effect of moral leadership on UPB. Moreover, we gain a better understanding of the paradoxical effects of positive leadership’s impact on UPB from the lens of identification. The current research either focuses on the dark side of positive leadership’s impact on UPB (Effelsberg et al., 2014; Kalshoven et al., 2016) or places the emphasis on the external guidance of positive leadership on UPB (Miao et al., 2013), we know little about how positive leadership shows its paradoxical effects by influencing subordinates’ intrinsic identification. Our study is an initial attempt to examine the paradoxical effects of positive leadership on UPB from the lens of identification. Third, we expand Social Identity Theory by incorporating social identity (identification with supervisors) and personal identity (taking responsibility) as mechanisms of the impact had by moral leadership upon UPB. Although social identity and personal identity provide different sources of meaning, Social Identity Theory says that social identity and personal identity are likely to work together on individual’s behavior, while we know little about how they work together (Stets and Burke, 2000). We further introduce moral courage as a personal characteristic boundary condition to explain when moral leadership decreases or increases UPB by mitigating or enhancing the impact of social identity or personal identity.

## Theory and Hypotheses

### Moral Leadership and Unethical Pro-organizational Behavior

Moral leadership is defined as “a leader’s behavior that demonstrates superior virtues, self-discipline, and unselfishness” (Cheng et al., 2004, p. 91). It entails “setting an example for others about the rightness or wrongness of particular actions” (Fairholm and Fairholm, 2009, p. 132), and exemplifies the exercise of integrity and fulfilling obligations, never taking advantage of others, and serving as a selfless paragon (Farh et al., 2008). With business scandals due to leaders’ lack of morality emerging endlessly, scholars reflected on the previous leadership research paying too much attention to leaders’ traits and behaviors while ignoring leaders’ morality (Kanungo and Mendonca, 1996). Moral leadership, which emphasizes leaders’ virtues, has received scholarly attention. Several studies have shown that moral leadership is positively related to positive employee behavior (Farh et al., 2006), such as organizational citizenship behavior (Tang and Naumann, 2015), which may be seen as ethical behavior (Effelsberg et al., 2014). Such forms of ethical behavior may also benefit the organization. That is to say, moral leadership is positively related to the ethical pro-organizational behavior. The question is raised about how moral leadership affects pro-organizational but unethical behaviors.

UPB refers to an action that can bring benefits to the organization but that is unethical because it violates societal ethical norms. For example, in order to improve the organizational reputation or maintain competitive advantage, employees exaggerate the accomplishments of their company (Cialdini et al., 2004). Umphress and Bingham (2011) indicate that UPB has two sides: the intent of the action—as employees intend to benefit their organization and the result of the action—as UPB is harmful to the interests of external stakeholders and society as a whole. Therefore, logically, if moral leadership makes employees behave ethically, it should decrease the unethical behavior even though the organization benefits. Meanwhile, if moral leadership truly triggers employees’ identification, it also should increase the pro-organizational behavior even though it is unethical.

Identification is often regarded as a key psychological mechanism explaining how leaders affect subordinates (Kark et al., 2003; Hobman et al., 2011). According to Social Identity Theory, identity is the core of the individual’s self-concept (Josselson, 1994); while identification reflects the extent to which one’s identity is based on group membership or individuated features (Tajfel, 1981; Ashforth and Mael, 1989). The high level of identification impels the individual to engage in activities consistent with his/her identity (Ashforth and Mael, 1989). Social Identity Theory contributes to explain why and how moral leadership is related to subordinates’ behavior.

Logically, moral leaders possess virtues, exercise responsibility, and set an example for others about what is right and wrong (Fairholm and Fairholm, 2009) and about cultivating concern for the common good (Tang and Naumann, 2015), which is likely to shape employees’ awareness of responsibility and ethical competence (Tang and Liu, 2012). Then “I’m a responsible person” becomes individual’s identity, which provides the moral foundation for action (Hackett and Wang, 2012). Thus, under moral leadership, employees may attach importance to the social moral standards and thus reduce UPB.

Meanwhile, intuitively, moral leaders demonstrate moral character and integrity by acting unselfishly (Cheng et al., 2004), who are highly respected and viewed as ideal leaders (Niu et al., 2009), which is likely to motivate subordinates to identify with and follow them (Kark et al., 2003; Gu et al., 2015). Then “I’m a subordinate of the leader” becomes individual’s identity, which motivates individual to act on behalf of leaders (Tajfel and Turner, 1986). Because leaders often represent the organization (Kalshoven and Den Hartog, 2009), moral leadership is likely to stimulate subordinates’ motivation to benefit the organization. The stronger subordinates identify with the leaders, the more likely they engage in UPB. Because such subordinates are eager to protect their identities, they probably put the interests of the leaders and organization above others’ (Kalshoven et al., 2016) and thus conceal the unethical information.

Therefore, we argue that the effect of moral leadership on UPB is likely to be a mixed impact. Next, we will explain how moral leadership increases or decreases UPB through identification with supervisors and taking responsibility.

### Mediating Role of Identification With Supervisors

Identification with supervisors is defined as subordinates’ perception of how their identities overlap with that of the supervisors (Li et al., 2015). This could be seen as the extent of the perceived oneness with supervisors (Ashforth et al., 2016). According to Social Identity Theory, identification with supervisors reflects the extent to which employees see themselves as a member of leaders (Tajfel, 1981; Ashforth and Mael, 1989). Moral leader behaves unselfishly and demonstrates moral character (Cheng et al., 2004) and that one is highly respected and viewed as an ideal leader (Niu et al., 2009), which raises the salience of the leader’s identity in subordinates’ self-concept, and frames followers’ roles of subordinates (Shamir et al., 1998; Johnson and Umphress, 2019). Therefore, moral leadership results in respect and identification from subordinates.

With an increase of identification with supervisors, employees perceive oneness with the leader, experience a psychologically entwinement with the leader, and share a common fate with the leader (Mael and Ashforth, 1992)—all of which illuminate the feeling of the belongingness of employees. Although research indicates that identification with supervisors is strongly related to employees’ positive behaviors, such as high performance (Becker et al., 1996) and organizational citizenship behaviors (Zhang and Chen, 2013), employees who highly identify with their supervisors will experience a deep affective and cognitive bond with the leader that shapes the extent to which the leader becomes a part of employees’ self-concept and that impels employees to support and benefit the leader (Tajfel and Turner, 1986). Because leaders represent the organization (Kalshoven and Den Hartog, 2009), employees may engage in UPB in order to contribute to the leader and the organization and thus ignore the unethical information of UPB. Moral leaders do not intentionally encourage UPB, while promoting the level of identification with the leader may *per se* accompany an increased subordinates’ intention to benefit the leader and the organization and even fall away the moral principle for the purpose. Therefore, we hypothesize the following:

**Hypothesis 1:** Identification with supervisors mediates the relationship between moral leadership and UPB.

### Mediating Role of Taking Responsibility

Taking responsibility is defined as individuals doing what they should do and being responsible for their actions (Ding et al., 2014). Hence, this concept depicts a behavioral feature of employees taking charge of what they did. According to Social Identity Theory, taking responsibility reflects the extent to which an individual’s self-concept is based on personal features (Tajfel, 1981). Thus, we argue that taking responsibility is a kind of personal identity that reflects the uniqueness of the individual (Brewer and Gardner, 1996). Deaux (1992) indicates that personal identity does not exist separately but on the basis of seeking the similarities with others. Moral leaders emphasize responsibility (Walker et al., 2007), exemplify for subordinates the distinction between rightness and wrongness of actions (Fairholm and Fairholm, 2009), and indicate ethical competence and moral courage (Haraway and Kunselman, 2006)—all of which are likely to influence subordinates through moral involvement (Etzoni, 1961), and thus evoke subordinates’ self-concept in the recognition that they share similar values with the leaders, or change subordinates’ self-concept so that their values become similar to that of the leaders (Pratt, 1998). Therefore, we argue that moral leadership is likely to enhance taking responsibility and thus promote the self-definition by subordinates as being responsible.

An employee with an increasing level of taking responsibility will show concern about and be responsible for his/her actions *per se* and consequences, which shows employee’s self-consistency (Swann et al., 1987). Further, in moral philosophy, what someone ought to do is always seen as a moral obligation, which refers to whether an individual has a moral reason to do it (Dill and Darwall, 2014). Thus, taking responsibility means a kind of moral judgment that provides employees with moral reason to do something or not. Research indicates that moral awareness, moral judgment, and moral disengagement are important determinants of whether behavior is ethical or unethical (Treviño et al., 2006; Putman, 2010). Therefore, we can safely infer that, due to explicit moral awareness, employees with high level of responsibility taking will attach importance to general moral standards and resist UPB in order to fulfill self-consistency and thus show that they are responsible persons, even though UPB benefits the organization. Taken together, moral leadership enhances the level of taking responsibility by shaping employees’ personal identity and thus reduces employees’ intention to engage in UPB. We thus hypothesize the following:

**Hypothesis 2:** Taking responsibility mediates the relationship between moral leadership and UPB.

### Moderating Effects of Moral Courage

The literature on Social Identity Theory posits that the impact of identity that is activated depends not only on situational factors but also on personal characteristics (Stets and Burke, 2000). A few studies have examined the effect of situational factors, such as interorganizational competition (Chen et al., 2016) and amoral culture (Umphress and Bingham, 2011), while some other studies have discussed the effect of personal characteristics, such as personal disposition toward ethical and unethical behavior (Effelsberg et al., 2014) and moral identity (Johnson and Umphress, 2019). As noted before, moral judgment is an important determinant of ethical and unethical behavior (Treviño et al., 2006; Putman, 2010). The moral judgment toward behavior includes both the commission of ethical behavior and restraint from unethical behavior (Bandura, 1999), that is, being ethical requires not only acting right but also inner fortitude when facing a moral dilemma (Monin et al., 2007). Moral courage is character strength in an individual who persists in personal moral principles and engages in ethical behavior. When experiencing moral dilemmas, such an individual commits to acting ethically based on his/her personal moral principles and resists pressure to influence his/her principles (Hannah et al., 2011). Moral courage invokes employees’ inner moral standards to act ethically (Sekerka and Bagozzi, 2007), which was found to be a critical factor in predicting whether employees act in line with their moral judgments (Hannah et al., 2011). Thus, we present that moral courage would be an important personal characteristic in effecting the impact of the identity beside personal disposition and moral identity.

As noted, identification with supervisors impels employees to intertwine psychologically with the leader, and promotes employee’s social identity—“I’m a subordinate of the leader” to be salient, which increases the probability of employees conducting UPB for benefiting the leader and the organization (Oakes, 1987). However, even in cases when employees have the same level of supervisor identification, they show different probabilities of conducting UPB. It depends on whether employees have the inner fortitude to act according to moral principle. Employees with high moral courage persist in moral standards and possess stronger psychological strength to face the tough ethical choices (e.g., Peterson and Seligman, 2004). Although employees with high moral courage may consider UPB as a kind of behavior that can bring benefit both to the leader and to the organization, they still address the moral dilemma between organizational benefit and moral principle and, thus, make the principled choice (Hannah and Avolio, 2010). Thus, high moral courage will weaken the positive relationship between identification with supervisors and UPB. On the contrary, employees with low moral courage exhibit difficulty in maintaining the inner moral standards and, thus, their supervisor identification will increase the probability of engaging in UPB for benefiting the leader and the organization. Further, it is moral leadership that shapes employees’ supervisor identification and thus increases the probability that employees conduct UPB. Therefore, high moral courage also weakens the mediated relationship between moral leadership and UPB through identification with supervisors. Thus, combined with H_1_, we propose the following hypothesis:

**Hypothesis 3_**a**_:** Moral courage moderates the relationship between identification with supervisors and UPB. That is to say, the positive relationship between supervisor identification and UPB is weaker for subordinates with high moral courage rather than those with low moral courage.

**Hypothesis 3_**b**_:** Moral courage moderates the mediated relationship between moral leadership and UPB through identification with supervisors. That is to say, the mediating effect is weaker for subordinates with high moral courage rather than those with low moral courage.

Employees who take responsibility know what they ought to do and consider it as a moral obligation, and thus promote their personal identity—“I’m a responsible person” to be salient, which increases the probability of them seeking rightful moral reasons for what they do and of making right moral judgment (Oakes, 1987). Employees with high moral courage persist in moral principle and act in line with moral judgment (Hannah et al., 2011), which intensifies the role of taking responsibility and enhances the impact of taking responsibility on UPB. Therefore, high moral courage strengthens the negative relationship between taking responsibility and UPB. In contrast, employees with low moral courage have difficulty in maintaining their inner moral standards, especially when encountering moral dilemma, they are likely to forgo their moral principle. Even though they define themselves as responsible persons, they have difficulty in maintaining their identities consistently. Therefore, for employees with low moral courage, the negative relationship between taking responsibility and UPB is weaker. Further, it is moral leadership that shapes employees’ taking responsibility and thus decreases the probability that employees conduct UPB. Therefore, high moral courage also strengthens the mediated relationship between moral leadership and UPB through taking responsibility. Combined with H_2_, we thus hypothesize the following:

**Hypothesis 4_**a**_:** Moral courage moderates the link between taking responsibility and UPB. That is to say, the negative association between taking responsibility and UPB is stronger for subordinates with high moral courage and weaker for those with low moral courage.

**Hypothesis 4_**b**_:** Moral courage moderates the mediated relationship between moral leadership and UPB through taking responsibility, such that the mediating effect is stronger for subordinates with high moral courage rather than those with low moral courage.

## Overview of Studies

We conducted two studies to test our hypotheses. Study 1 was conducted with Master of Business Administration (MBA) students in China. Although nearly all hypotheses were supported in the MBA student sample, we anticipated that the research results could be generalized to business organizations. In addition, we expected that the self-developed measure for taking responsibility could be verified again in a new sample. Therefore, we conducted Study 2 to attempt to replicate the results of Study 1 in a business and to provide stronger evidence for the self-developed measure. Study 2 was conducted in a trading company located in a southern province of China, which provided stronger evidence for generalizability and for our new scale of taking responsibility. Together, the two studies evaluated our research model through field methodologies, which strengthened the ecological validity of our conclusions.

## Materials and Methods

### Scale Development for Taking Responsibility

There is no widely used measure for taking responsibility. We review the literature on responsibility, obligation, and accountability and find there are a few studies that have addressed the responsibility that a person should take for what that person does. The core component of responsibility is holding people accountable for their conduct (Schlenker et al., 1994). For this study, we define “taking responsibility” as a person undertaking duties and being accountable for his actions (Ding et al., 2014). The connotation of taking responsibility contains three levels. First, people are responsible for each act that is connected with them (c.f. Schlenker et al., 1994), including not only their duties but also anything they identifies with. Second, people are bold in taking on and trying their best to do each act what they are responsible for. Third, if they cannot fulfill their responsibilities, they are responsible for the effects of any act they committed (c.f. Schlenker et al., 1994). Despite the overlaps, taking responsibility is conceptually distinct from other constructs, such as responsibility acceptance (Pace et al., 2010; Wenzel et al., 2012), felt obligation (Eisenberger et al., 2001), holding oneself accountable (Dill and Darwall, 2014), conscientiousness (Roberts et al., 2012), and taking charge (Morrison and Phelps, 1999). Please see Table 1 for a detailed summary of the differences between taking responsibility and other constructs.

**TABLE 1:** Summary of similarity and differences between taking responsibility and other related constructs.

	**Taking Responsibility**	**Responsibility Acceptance**	**Felt Obligation**	**Taking Charge**	**Holding Oneself Accountable**	**Conscientiousness**
Association	Each act that belongs to a person’s duty or a person identifies with	One’s wrongful action	The organization’s welfare and objectives	Organizationally functional change (extra-role behavior)	One’s wrongful action	Promises to others and rules that make social work more smoothly
Commission	The effects of any act he/she committed	Accepting responsibility for the harm caused	Uninvolved	Uninvolved	Accepting punishment or sanction for one’s wrongdoing	Uninvolved
Morality related	√	√	Uninvolved	Uninvolved	√	Uninvolved
Active or passive	Active	Passive	Active	Active	Both	Active
Cognition-action combination	√	Cognition	Cognition	Action	√	√
*Ex ante* or *ex post* responsibility	Both	*Ex post*	Uninvolved	Uninvolved	*Ex post*	Uninvolved
Heteronomy or autonomy	Autonomy	Heteronomy	Heteronomy	Autonomy	Heteronomy	Autonomy
Emotion related	Uninvolved	√	√	Uninvolved	√	Uninvolved
Context	General	Legal context	Organizational context	The contexts of job, work unit, or organizations	Legal context	General
Directionality	General	To others	To organization	To job, work unit, or organization	To others	To others

*√ means the presence of the corresponding component in the construct.*

On the basis of the literature on responsibility, we generated six items to describe taking responsibility. We then invited two leadership researchers to assess these items and improved their clarity and accuracy, which to some extent ensured the facial validity of the construct.

We invited another four organizational behavior researchers, who were outside our research team and not aware of our research purpose. Of four experts, three were professors, and one was an assistant professor. The four experts all majored in leadership and possessed Ph.D. degrees. We informed the four experts about the meanings of four constructs: taking responsibility, responsibility acceptance, felt obligation, and taking charge. Then we demonstrated six items of taking responsibility to four experts and required them to match six items to the suitable construct. The results indicated that there was high inter-judge raw agreement (average raw agreement = 1) and high placement ratio of items within the target construct (average placement ratio = 1). On the basis of matching items to the suitable construct, we asked four experts to rate the matching degree of six items on an 8-point Likert scale (1 = “not at all,” 8 = “fully matching”). Then, we calculated the coefficient of Cohen’s Kappa (average κ = 0.66), which was high above 0.61 and indicated that the agreement between four raters was substantial (Cohen, 1960; Landis and Koch, 1977). These results provided evidence not only of the content validity but also of the convergent validity and discriminant validity of the construct of taking responsibility (Moore and Benbasat, 1991).

We recruited one sample to assess the item quality of taking responsibility and used another two samples to assess the validity of the scale. Hundred and twelve MBA students were recruited online through Wechat, who were all from a university in Beijing. They understood our research purpose and participated in our survey voluntarily. At last, we received 80 valid responses. The proportion of female respondents in our sample was 66.3 percent. Of these participants, 71.3 percent were married. Most of the participants, about 82.5 percent, had a bachelor’s degree. Around 46.3 percent of participants were in non-management positions, and 51.3 percent were in managerial roles. The participants’ average age was 33.9 years(*SD* = 4.36), the average working years was 11.4 years (*SD* = 4.41), and the average tenure in their present organization was 4.88 years (*SD* = 5.00).

We conducted Critical Ratio, Corrected Item-Total Correlation, and Exploratory Factor Analysis to validate the items. We deleted the item if it did not pass any one of the tests. At last, we retained three items (see Appendix). As shown in Table 2, the Critical Ratio values of the three items are all significant (*p* < 0.001). The Corrected Item-Total Correlation values of the three items were all greater than 0.5 (ranging from 0.603 to 0.613) (Nunnally, 1978), which indicated that the three items were related to the total score for taking responsibility. The results of Kaiser-Meyer-Olkin (KMO = 0.701) and Bartlett’s Test of Sphericity (*p* < 0.001) indicated that the scale for taking responsibility was suitable for factor analysis. Using the principal component analysis to extract Eigenvalue factors greater than 1, we extracted one factor, which accounted for 68.849 percent of the variance. The three items’ standardized loadings on taking responsibility exceeded 0.7 (ranging from 0.720 to 0.741). These results showed that the three items retained to measure taking responsibility have passed the three kinds of tests mentioned above. We then tested composite reliability of the scale, which was 0.774, greater than the psychometric standard (>0.70).

**TABLE 2:** Results of Critical Ratio significance, Corrected Item-Total Correlation and Factor Loading about items of taking responsibility.

**Items**	**Critical Ratio Significance**	**Corrected Item-Total Correlation**	**Factor Loading**
It is my obligation to be responsible for the scope of my duties	0.000	0.605	0.728
I would be responsible for my mistake	0.000	0.613	0.741
I would not defer responsibility to others when I did something wrong	0.000	0.603	0.720
Eigenvalue			2.065
Cumulative variance%			68.849

**N* = 80.*

We recruited another sample to assess the validity of taking responsibility. Two hundred and fifteen employees were recruited online through Wechat from five companies in China. They all understood our research purpose and participated in our survey voluntarily. We received 215 valid responses. The proportion of female respondents in our sample was 64.7 percent. Of these participants, 67.9 percent were married. Most of the participants, about 66.1 percent, had a bachelor’s degree. Around 71.9 percent of participants were in non-management positions, and 19.5 percent were first-line managers. The participants’ average age was 33.8 years (*SD* = 7.66), their average working years was 9.74 years (*SD* = 7.88).

We performed a series of Confirmatory Factor Analyses (CFA) using Mplus 7.4. An excellent fit was found for three items rather than six items (χ^2^ = 40.188, df = 19, χ^2^/df = 2.115, RMSEA = 0.071, CFI = 0.980, TLI = 0.970), with all three items strongly loading on taking responsibility (ranging from 0.658 to 0.889). Additionally, as in Table 3, three two-factor models fit the data significantly better than other three one-factor models. Further, the variable of taking responsibility had the desired convergent validity because the standard factor loadings of each item on taking responsibility (ranging from 0.658 to 0.889) were larger than 0.5 and the AVE of taking responsibility was 0.653, which was larger than 0.5 (Hair et al., 2006). The variable of taking responsibility also had the desired discriminant validity because its AVE was larger than the squared correlations between taking responsibility and responsibility acceptance (*r* = 0.465, *r*^2^ = 0.216), felt obligation (*r* = 0.737, *r*^2^ = 0.543), and taking charge (*r* = 0.488, *r*^2^ = 0.238) (Hair et al., 2006).

**TABLE 3:** Results of CFA about taking responsibility and other related constructs.

		**χ^2^**	**df**	**χ^2^/df**	**RMSEA**	**CFI**	**TLI**	**Δχ^2^**
Taking responsibility and felt obligation	Two-factor model	40.188	19	2.115	0.071	0.980	0.970	
	One-factor model	156.123	20	7.806	0.175	0.869	0.816	115.935^∗∗∗^
Taking responsibility and responsibility acceptance	Two-factor model	70.693	33	2.142	0.072	0.956	0.940	
	One-factor model	293.419	34	8.630	0.186	0.696	0.598	228.597^∗∗∗^
Taking responsibility and taking charge	Two-factor model	136.723	57	2.399	0.080	0.960	0.945	
	One-factor model	370.286	58	6.384	0.156	0.843	0.789	233.563^∗∗∗^

**N* = 215, ^∗∗∗^*p* < 0.001.*

Additionally, we used the sample in Study 2 to verify the convergent validity and discriminant validity of taking responsibility again with the AVE method. The results indicated that the standard factor loadings of each item on taking responsibility (ranging from 0.599 to 0.926) were larger than 0.5 and the AVE of taking responsibility was 0.674, which was larger than 0.5. The variable of taking responsibility also had the desired discriminant validity because its AVE was larger than the squared correlations between taking responsibility and moral leadership (*r* = 0.233, *r*^2^ = 0.054), identification with supervisors (*r* = 0.266, *r*^2^ = 0.071), moral courage (*r* = 0.124, *r*^2^ = 0.015), and UPB (*r* = −0.215, *r*^2^ = 0.046).

To provide further evidence about the taking responsibility measurement, we also took UPB as a criterion variable of taking responsibility in formal study. In order to validate the criterion-related validity of taking responsibility, we used the correlation and grouping methods. Taking responsibility was significantly related to UPB (*r*_sample__1_ = −0.243, *p* < 0.01; *r*_sample__2_ = −0.215, *p* < 0.01). We divided UPB into high (+1 standard deviation) and low score groups (−1 standard deviation). Through a *T*-test analysis, we found that there was a significant difference between high score and low score groups in taking responsibility (*t*_sample__1_ = 3.863, *p* < 0.001; *t*_sample__2_ = 2.958, *p* < 0.01). After accounting for the control variables (gender, marital status, position, educational background, organization type, age, working years, and organizational tenure), taking responsibility was negatively related to UPB (β_sample__1_ = −0.300, *p* < 0.01; β_sample__2_ = −0.304, *p* < 0.01). These results showed that the criterion-related validity of taking responsibility was accepted. The aforementioned scale development procedures and the empirical results supported our conceptualization of taking responsibility.

### Measurements

All measurements were rated on a 6-point Likert scale (1 = “strongly disagree” to 6 = “strongly agree”). Furthermore, we also collected demographic variables. The scales from the original English versions were translated from English to Chinese and back to English using translation and back-translation procedures (Brislin, 1986).

#### Moral Leadership

Using a 5-item scale developed by Cheng et al. (2000), we asked respondents to rate the moral leadership of their immediate supervisors. A sample item was, “He is impartial to everyone.” Composite reliabilities (CRs) for sample 1 and sample 2 were 0.929 and 0.927, respectively.

#### Identification With Supervisor

Identification with supervisor was measured using a 7-item scale developed by Shamir et al. (1998). Respondents were asked to rate the degree to which they identified with their immediate supervisors. A sample item was, “I have complete faith in my supervisor.” CR was 0.921 for sample 1 and 0.924 for sample 2.

#### Taking Responsibility

The 3-item scale developed for this study was used to measure taking responsibility. CR for the taking responsibility scale was 0.871 for sample 1 and 0.857 for sample 2.

#### Moral Courage

Moral courage was assessed by respondents on the basis of a 4-item scale developed by Hannah et al. (2011). A sample item was, “Confront my peers if they commit an unethical act.” CRs for sample 1 and sample 2 were 0.839 and 0.769, respectively.

#### Unethical Pro-organizational Behavior

UPB was measured using a 6-item scale adopted from Umphress et al. (2010). Respondents were asked to rate UPB in a self-reported questionnaire. A sample item was, “If needed, I would conceal information from the public that could be damaging to my organization.” CR was 0.790 for sample 1 and 0.772 for sample 2.

#### Control Variables

As prior research has shown that demographic characteristics may affect the extent to which individuals take part in unethical behavior (Erdogan and Liden, 2002), we include demographic characteristics, such as age, gender, marital status, educational background, years of work experience, organizational tenure, the position in an organization, and organizational type in our study to control for their potential effects.

### Analytical Procedures

The data analysis was undertaken using SPSS25.0 and Mplus7.4 in three steps. First, a series of Confirmatory Factor Analyses (CFAs) were conducted with Mplus7.4 to test the discriminant validity of the variables.

Second, we tested possible common method variance *via* the unmeasured latent method construct (ULMC) technique (Podsakoff et al., 2003).

Third, we tested all the research hypotheses using Mplus 7.4. We evaluated the research model (Figure 1) and an alternative model (adding the direct path from moral leadership to UPB based on the research model), and then we chose the optimal model for hypothesis testing. Then we used the bias-corrected bootstrapping method through 10000 resamples to test mediation effects and moderated mediation effects because the bootstrapping method overcomes the problem of non-normality distribution and estimates indirect effects more accurately (Preacher and Hayes, 2008).

## Study 1

### Participants and Procedure

To test our hypotheses, we recruited MBA students from a university in Shanxi Province and collected data in two phases. Before recruiting participants, we received the approval of university principal. Participation in our study was entirely voluntary. Surveys were distributed to individuals and then returned on the spot. To protect the privacy of participants, all responses were anonymous. We gave each MBA student a sealed envelope with an informed consent form and two questionnaires and a book named Zero to One as a gift. Questionnaires were administered to 176 MBA students. In phase 1, we asked MBA students to rate their moral courage and the moral leadership of their immediate supervisors. In phase 2 (3 h later), we collected identification with supervisors, taking responsibility, and UPB. Of the 176 questionnaires, 161 were valid, and 15 were eliminated. The valid response rate was 91.48 percent.

Of these participants, 56.5 percent were female and 59.6 percent were married. The proportion of recipients with a bachelor’s degree was 41.0 percent. Among these MBA students, 46.6 percent were first-line managers and 32.3 percent were ordinary staff. Participants were from several types of organizations (e.g., 50.3 percent of them were from state-owned enterprises and 13.7 percent were from private enterprises). On average, participants were 28.42 years (*SD* = 2.717) old, had been in their professions for around 5.30 years (*SD* = 2.598), and had been employed in their organizations for approximately 4.16 years (*SD* = 2.358).

### Results

#### Confirmatory Factor Analyses

We conducted Confirmatory Factor Analysis (CFA) using Mplus 7.4 to evaluate the variables’ distinctness. We compared the goodness-of-fit indices of the five-factor model with other nested models. As shown in Table 4, the CFA results demonstrated that our hypothesized five-factor model was a better fit into the data (χ^2^ = 301.060, df = 199, χ^2^/df = 1.513, RMSEA = 0.057, CFI = 0.948, TLI = 0.940) than the more parsimonious four-factor (χ^2^ = 560.499, df = 203, χ^2^/df = 2.761, RMSEA = 0.106, CFI = 0.819, TLI = 0.794), three-factor (χ^2^ = 809.634, df = 206, χ^2^/df = 3.930, RMSEA = 0.137, CFI = 0.695, TLI = 0.658), two-factor (χ^2^ = 1030.151, df = 208, χ^2^/df = 4.953, RMSEA = 0.159, CFI = 0.585, TLI = 0.539), and one-factor(χ^2^ = 1231.338, df = 209, χ^2^/df = 5.892, RMSEA = 0.177, CFI = 0.484, TLI = 0.429) models, which showed that these five variables in our study could be discriminated from each other.

**TABLE 4:** Results of confirmatory factor analysis (Study 1).

**Model**	**χ^2^**	**df**	**χ^2^/df**	**RMSEA**	**CFI**	**TLI**	**Δχ^2^**
Five-factor model	301.060	199	1.513	0.057	0.948	0.940	
Four-factor model	560.499	203	2.761	0.106	0.819	0.794	259.439^∗∗∗^
Three-factor model	809.634	206	3.930	0.137	0.695	0.658	508.574^∗∗∗^
Two-factor model	1030.151	208	4.953	0.159	0.585	0.539	729.091^∗∗∗^
One-factor model	1231.338	209	5.892	0.177	0.484	0.429	930.278^∗∗∗^

**N* = 161, ^∗∗∗^*p* < 0.001; Five-factor model: Moral leadership, identification with supervisors, taking responsibility, moral leadership, UPB; Four-factor model: Identification with supervisors + taking responsibility; Three-factor model: Identification with supervisors + taking responsibility + moral courage; Two-factor model: Moral leadership + identification with supervisors + taking responsibility + moral courage; One-factor model: Moral leadership + identification with supervisors + taking responsibility + moral courage + UPB.*

#### Common Method Bias (CMB) Testing

In Study 1, the study variables were all assessed by the MBA students themselves’ which made a CMB result possible. We conducted the unmeasured latent method construct (ULMC) approach to confirm whether the CMB was serious or not. We added a single unmeasured latent method factor to the baseline model (i.e., five-factor model). Compared to the fit of the baseline model, the goodness-of-fit indices of the latent method factor model (χ^2^ = 295.899, df = 193, χ^2^/df = 1.533, RMSEA = 0.058, CFI = 0.948, TLI = 0.938) did not improve significantly [Δχ*^2^* (6) = 5.161, *p* > 0.05). The result demonstrated that CMB was not a serious problem.

#### Descriptive Statistics and Correlations

The means, standard deviations, bivariate correlations among study variables, and the squared root of AVE for each scale are presented in Table 5. The correlations showed that moral leadership was positively related to identification with supervisors (*r* = 0.654, *p* < 0.001), identification with supervisors was positively related to UPB (*r* = 0.248, *p* < 0.01), moral leadership was positively related to taking responsibility (*r* = 0.281, *p* < 0.001), and taking responsibility was negatively related to UPB (*r* = −0.243, *p* < 0.01). However, moral leadership did not significantly correlate with UPB (*r* = 0.031, *ns*), indicating both that moral leadership may have positive and negative effects on UPB and that the two effects may neutralize each other. This offered preliminary support for our theoretical hypotheses. Moreover, the composite reliabilities of the five scales all exceeded 0.7, and the squared root of AVE for each scale was higher than the construct’s respective correlation with other constructs (Fornell and Larcker, 1981), indicating the psychometric properties of five scales acceptable.

**TABLE 5:** Means, standard deviations, correlations among constructs (Study 1).

**Variables**	***M***	***SD***	**1**	**2**	**3**	**4**	**5**
(1) Moral leadership	4.16	1.020	(0.850)				
(2) Identification with supervisors	3.99	0.930	0.654^∗∗∗^	(0.791)			
(3) Taking responsibility	5.19	0.655	0.281^∗∗∗^	0.173^∗^	(0.834)		
(4) Moral courage	3.48	0.862	0.243^∗∗^	0.275^∗∗∗^	0.125	(0.758)	
(5) Unethical pro-organizational behavior	3.25	0.811	0.031	0.248^∗∗^	–0.243^∗∗^	−0.157^∗^	(0.628)

**N* = 161, ^∗^*p* < 0.05, ^∗∗^*p* < 0.01, ^∗∗∗^*p* < 0.001, two-tailed tests; Square root of the average variance extracted values are given in parentheses on the diagonal.*

#### Hypothesis Testing

We performed Structural Equation Modeling (SEM) using Mplus 7.4 to test our hypotheses. Based on our research model, we developed an alternative model by adding a direct path from moral leadership to UPB in order to choose the optimal model. The results indicated that both the research model (χ^2^ = 349.928, df = 251, χ^2^/df = 1.394, RMSEA = 0.050, CFI = 0.941, TLI = 0.931) and the alternative model (χ^2^ = 346.893, df = 250, χ^2^/df = 1.388, RMSEA = 0.050, CFI = 0.943, TLI = 0.932) fit the data well. However, the chi-square change was not significant [Δχ^2^ (1) = 3.035, *p* > 0.05], showing that the alternative model did not significantly improve the research model. Based on the principle of model parsimony, we chose the research model for our hypothesis testing.

##### Testing H_1_

We attained results showing both that moral leadership was positively related to identification with supervisors (β = 0.692, *p* < 0.001, BC bootstrap 95% CI = [0.508,0.827], excluding 0) and that identification with supervisors was positively related to UPB (β = 0.334, *p* < 0.001, BC bootstrap 95% CI = [0.165,0.512], excluding 0) after controlling for demographic variables (gender, marriage, age, education, position, organizational type, working years and organizational tenure). The mediating effect of identification with supervisors upon the relationship between moral leadership and UPB was significant (indirect effect = 0.232, *p* < 0.001, BC bootstrap 95% CI = [0.128,0.365], excluding 0). These findings provided support for H_1_.

##### Testing H_2_

The results showed both that moral leadership was positively related to taking responsibility (β = 0.338, *p* < 0.001, BC bootstrap 95% CI = [0.183,0.497], excluding 0) and that taking responsibility was negatively related to UPB (β = −0.371, *p* < 0.001, BC bootstrap 95% CI = [−0.541, −0.176], excluding 0) after controlling for demographic variables (gender, marriage, age, education, position, organizational type, working years and organizational tenure). The mediating effect of taking responsibility upon the relationship between moral leadership and UPB was significant (indirect effect = −0.125, *p* < 0.01, BC bootstrap 95% CI = [−0.228, −0.055], excluding 0). These findings provided support for H_2_.

##### Testing H_3__*a*_ and H_4__*a*_

**FIGURE 2:**
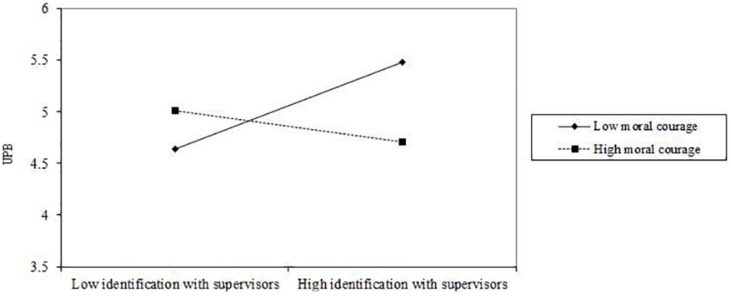
The hypothetical moderating effect of moral courage on the relationship between identification with supervisors and UPB in Study 1.

We used a single product indicant to create the interaction term (Ping, 1995) in the structural model. After adding the interaction terms to the mediation model, we found that the model also fit the data well (χ^2^ = 470.278, df = 309, χ^2^/df = 1.522, RMSEA = 0.059, CFI = 0.916, TLI = 0.902). The results showed that moral courage moderated the effect of identification with supervisors on UPB (β = −0.385, *p* < 0.001, BC bootstrap 95% CI = [−0.568, −0.194], excluding 0) such that the positive relationship between identification with supervisors and UPB was weaker for employees with high rather than low moral courage. We calculated one standard deviation above and below the mean to plot the interaction effects (Aiken and West, 1991). Figure 2 illustrated the moderating effect of moral courage on the relationship between identification with supervisors and UPB. Thus, H_3__a_ was supported in sample 1. However, moral courage did not moderate the relationship between taking responsibility and UPB (β = −0.080, *p* > 0.05, BC bootstrap 95% CI = [−0.245,0.093], including 0). Therefore, H_4__a_ was not supported in sample 1.

##### Testing H_3__*b*_ and H_4__*b*_

We adopted a moderated mediation analytic procedure proposed by Edwards and Lambert (2007) to test hypothesis 3b and 4b. For the group with high and low moral courage, the indirect effect of moral leadership on UPB through identification with supervisors was significantly different (β = −0.144, *p* < 0.001, BC bootstrap 95% CI = [−0.244, −0.073], excluding 0). As shown in Table 6, when moral courage was low, the indirect effect of moral leadership on UPB through identification with supervisors was significant (indirect effect = 0.313, *p* < 0.001, BC bootstrap 95% CI = [0.173,0.521], excluding 0). However, when moral courage was high, the indirect effect of moral leadership on UPB through identification with supervisors was not significant (indirect effect = 0.065, *p* > 0.05, BC bootstrap 95% CI = [−0.089,0.241], including 0). The difference of the indirect effects between the group with low and high moral courage was significant (Δ indirect effect = 0.248, *p* < 0.001, BC bootstrap 95% CI = [0.125,0.421], excluding 0). Thus, H_3__b_ was supported.

**TABLE 6:** Summary of moderated mediation (Study 1).

**Moderator variable**	**Moral leadership (X) → identification with supervisors (M) → unethical pro-organizational behavior (Y)**		
	
	**First stage (P_MX_)**	**Second stage (P_*YM*_)**	**Indirect effects (P_MX_^∗^ P_YM_)**
Low moral courage (−1 SD)	0.558^∗∗∗^	0.561^∗∗∗^	0.313^∗∗∗^ (95% CI = [0.173,0.521])
High moral courage (+ 1 SD)	0.558^∗∗∗^	0.117	0.065 (95% CI = [−0.089,0.241])
Differences	0	0.444^∗∗∗^	0.248^∗∗∗^ (95% CI = [0.125,0.421])

**Moderator variable**	**Moral leadership (X) → taking responsibility (M) →unethical pro-organizational behavior (Y)**		
	
	**First stage (P_MX_)**	**Second stage (P_*YM*_)**	**Indirect effects (P_MX_^∗^ P_YM_)**

Low moral courage (−1 SD)	0.188^∗∗∗^	–0.163	−0.031 (95% CI = [−0.086,0.012])
High moral courage (+ 1 SD)	0.188^∗∗∗^	−0.296^∗^	−0.056^†^(95% CI = [−0.136,−0.012])
Differences	0	0.133	0.025 (95% CI = [−0.022,0.106])

**N* = 161, ^†^*p* < 0.1, ^∗^*p* < 0.05, ^∗∗∗^*p* < 0.001; bootstrap = 10000. Unstandardized path coefficients are reported. P_*MX*_, first-stage effect of predictor X on mediator (M); P_*YM*_, second-stage effect of M on Y. Tests of P_*MX*_, P_*YM*_, and P_*MX*_^∗^ P_*YM*_ are based on bias-corrected confidence intervals derived from bootstrap estimates.*

However, for the group with high and low moral courage, the indirect effect of moral leadership on UPB through taking responsibility was not significantly different (β = −0.014, *p* > 0.05, BC bootstrap 95% CI = [−0.061,0.013], including 0). As shown in Table 6, when moral courage was low, the indirect effect of moral leadership on UPB through taking responsibility was not significant (indirect effect = −0.031, *p* > 0.05, BC bootstrap 95% CI = [−0.086,0.012], including 0). When moral courage was high, the indirect effect of moral leadership on UPB through taking responsibility was significant (indirect effect = −0.056, *p* < 0.1, BC bootstrap 95% CI = [−0.136, −0.012], excluding 0). Yet the difference of the indirect effects between the group with low and high moral courage was not significant (Δ indirect effect = 0.025, *p* > 0.05, BC bootstrap 95% CI = [−0.022,0.106], including 0). Thus, H_4__b_ was not supported in sample 1.

In Study 1, we tested our hypotheses in a sample of MBA students and found that moral leadership affected UPB *via* identification with supervisors and taking responsibility, and moral courage moderated the mediating effect of moral leadership on UPB through identification with supervisors. However, the moderating effect of moral courage on the mediating effect of taking responsibility upon the relationship between moral leadership and UPB was not supported in sample 1. Although Study 1 provides preliminary empirical evidence for our research model, it was important to note that our findings were only limited to the MBA student sample. We thus sought to address this limitation in Study 2, in which we attempted to generalize the results of Study 1 to an employee sample.

## Study 2

### Participants and Procedure

In this study, we collected two waves of survey data, 1 month apart from each other, from a trading company located in the southern province of China. To apply for participation, we gave a presentation about the purpose and benefits of our research to the company. With strong support from the company, we conducted a two-wave data collection based on our research model. In each phase, surveys were distributed to individuals and then returned on the spot. We gave each individual a sealed envelope with an informed consent form and a questionnaire. Participation in our study was entirely voluntary. To protect the privacy of participants, all responses were anonymous. To match two-phase data, we asked participants to write down a special code with his/her mother’s last name and last four digits of his/her cell phone number.

In phase 1, questionnaires were sent to 252 employees, who were requested to rate their moral courage and the moral leadership of their immediate supervisors. We received 238 responses, yielding a response rate of 94.4 percent. One month later, we engaged in phase 2 data collection. In phase 2, we collected data upon identification with supervisors, taking responsibility, and UPB. Surveys were administered to 239 employees, because 13 of the 252 employees in phase 1 took turns taking holiday. Among them, 217 responded to our survey, yielding a response rate of 90.5 percent. We filtered the questionnaires that were unmatched. After filtering, 205 responses were valid, reaching a valid response rate of 94.5%.

Of these participants, 64.4 percent were female, and 62.9 percent were married. The proportion of junior college and bachelor’s degree recipients was 41.0 percent and 24.4 percent, respectively. Among these employees, 24.9 percent of participants were first-line managers, and 62.9 percent of them were ordinary staff. On average, participants were 35.71 years (*SD* = 9.924) old, had been in their professions for around 14.29 years (*SD* = 9.993), and had been employed in their organizations for approximately 5.48 years (*SD* = 5.048).

### Results

#### Confirmatory Factor Analyses

As in Study 1, we conducted a series of CFAs to evaluate the variables’ distinctness. As shown in Table 7, the CFA results demonstrated that our hypothesized five-factor model fit the data (χ^2^ = 395.641, df = 199, χ^2^/df = 1.988, RMSEA = 0.071, CFI = 0.920, TLI = 0.907) better than the more parsimonious four-factor (χ^2^ = 699.728, df = 203, χ^2^/df = 3.447, RMSEA = 0.112, CFI = 0.799, TLI = 0.771), three-factor (χ^2^ = 902.960, df = 206, χ^2^/df = 4.383, RMSEA = 0.132, CFI = 0.717, TLI = 0.683), two-factor (χ^2^ = 1138.144, df = 208, χ^2^/df = 5.472, RMSEA = 0.152, CFI = 0.623, TLI = 0.581), and one-factor (χ^2^ = 1710.877, df = 209, χ^2^/df = 8.186, RMSEA = 0.192, CFI = 0.391, TLI = 0.327) models, which showed that these five variables could be discriminated from each other.

**TABLE 7:** Results of confirmatory factor analysis (Study 2).

**Model**	**χ^2^**	**df**	**χ^2^/df**	**RMSEA**	**CFI**	**TLI**	**Δχ^2^**
Five-factor model	395.641	199	1.988	0.071	0.920	0.907	
Four-factor model	699.728	203	3.447	0.112	0.799	0.771	304.087^∗∗∗^
Three-factor model	902.960	206	4.383	0.132	0.717	0.683	507.319^∗∗∗^
Two-factor model	1138.144	208	5.472	0.152	0.623	0.581	742.503^∗∗∗^
One-factor model	1710.877	209	8.186	0.192	0.391	0.327	1315.236^∗∗∗^

**N* = 205, ^∗∗∗^*p* < 0.001; Five-factor model: Moral leadership, identification with supervisors, taking responsibility, moral leadership, UPB; Four-factor model: Identification with supervisors + taking responsibility; Three-factor model: Identification with supervisors + taking responsibility + moral courage; Two-factor model: Moral leadership + identification with supervisors + taking responsibility + moral courage; One-factor model: Moral leadership + identification with supervisors + taking responsibility + moral courage + UPB.*

#### Common Method Bias (CMB) Testing

In Study 2, the study variables were also evaluated by the employees themselves, which facilitated a CMB result. Therefore, we also conducted ULMC technique to confirm whether the CMB was serious or not. According to Cheung and Rensvold (2002), when the sample size exceeds 200, ΔCFI is superior to Δχ^2^ as a test of invariance because it is not affected by sample size. When the value of ΔCFI is smaller than 0.01, the alternative model cannot be significantly better than the baseline model. After adding a single unmeasured latent method factor to the baseline model, the goodness-of-fit indices for the latent method factor model did not improve significantly (ΔCFI = 0.009, *p* < 0.01), which demonstrated that the CMB was not a serious problem.

#### Descriptive Statistics and Correlations

The means, standard deviations, bivariate correlations among study variables, and the squared root of AVE for each scale are presented in Table 8. The correlations showed that moral leadership was positively related to identification with supervisors (*r* = 0.464, *p* < 0.001), identification with supervisors was positively related to UPB (*r* = 0.208, *p* < 0.001), moral leadership was positively related to taking responsibility (*r* = 0.233, *p* < 0.001), and taking responsibility was negatively related to UPB (*r* = −0.215, *p* < 0.01). However, moral leadership did not significantly correlate with UPB (*r* = 0.062, *ns*), which indicated both that moral leadership may have positive and negative effects on UPB and that the two effects may neutralize each other. Moreover, the composite reliabilities of the five scales all exceeded 0.7, and the squared root of AVE for each scale was higher than the construct’s respective correlation with other constructs (Fornell and Larcker, 1981), indicating an acceptable level of psychometric properties of five scales.

**TABLE 8:** Means, standard deviations, correlations among constructs (Study 2).

**Variables**	***M***	***SD***	**1**	**2**	**3**	**4**	**5**
(1) Moral leadership	4.90	0.846	(0.848)				
(2) Identification with supervisors	4.59	0.838	0.464^∗∗∗^	(0.797)			
(3) Taking responsibility	5.30	0.556	0.233^∗∗∗^	0.266^∗∗∗^	(0.821)		
(4) Moral courage	4.21	0.850	0.169^∗^	0.302^∗∗∗^	0.124	(0.689)	
(5) Unethical pro-organizational behavior	3.31	0.882	0.062	0.208^∗∗^	–0.215^∗∗^	0.019	(0.607)

**N* = 205, ^∗^*p* < 0.05, ^∗∗^*p* < 0.01, ^∗∗∗^*p* < 0.001, two-tailed tests; Square root of the average variance extracted values are given in parentheses on the diagonal.*

#### Hypothesis Testing

As in Study 1, we developed an alternative model by adding a direct path from moral leadership to UPB in order to choose the optimal model. The result indicated that both the research model (χ^2^ = 195.937, df = 131, χ^2^/df = 1.496, RMSEA = 0.052, CFI = 0.959, TLI = 0.947) and the alternative model (χ^2^ = 195.553, df = 130, χ^2^/df = 1.504, RMSEA = 0.053, CFI = 0.959, TLI = 0.946) fit the data well. However, the chi-square change was not significant [Δχ^2^ (1) = 0.384, *p* > 0.05]. Based on the principle of model parsimony, we chose the research model for our hypothesis testing.

##### Testing H_1_

We attained results showed both that moral leadership was positively related to identification with supervisors (β = 0.515, *p* < 0.001, BC bootstrap 95% CI = [0.374,0.640], excluding 0) and that identification with supervisors was positively related to UPB (β = 0.250, *p* < 0.01, BC bootstrap 95% CI = [0.076,0.414], excluding 0) after controlling for demographic variables (gender, marriage, age, education, position, working years, and organizational tenure). The mediating effect of identification with supervisors upon the relationship between moral leadership and UPB was significant (indirect effect = 0.129, *p* < 0.01, BC bootstrap 95% CI = [0.047,0.227], excluding 0). Thus, H_1_ was supported.

##### Testing H_2_

We received results showed both that moral leadership was positively related to taking responsibility (β = 0.241, *p* < 0.01, BC bootstrap 95% CI = [0.058,0.410], excluding 0) and that taking responsibility was negatively related to UPB (β = −0.240, *p* < 0.01, BC bootstrap 95% CI = [−0.415,−0.067], excluding 0) after controlling for demographic variables (gender, marriage, age, education, position, working years, and organizational tenure). The mediating effect of taking responsibility upon the relationship between moral leadership and UPB was significant (indirect effect = −0.058, *p* < 0.01, BC bootstrap 95% CI = [−0.144,−0.011], excluding 0). These findings provided support for H_2_.

##### Testing H_3__*a*_ and H_4__*a*_

**FIGURE 3:**
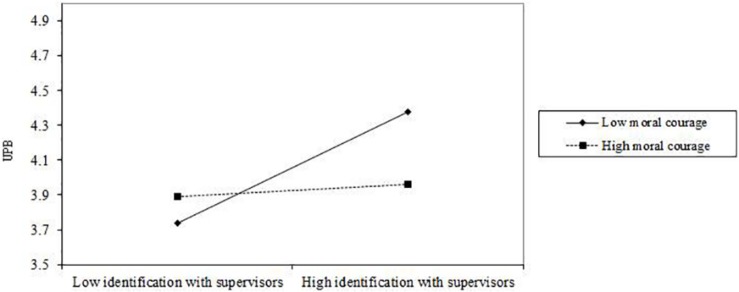
The hypothetical moderating effect of moral courage on the relationship between identification with supervisors and UPB in Study 2.

As in Study 1, we used a single product indicant to create the interaction term (Ping, 1995) in the structural model and added the interaction terms to the mediation model. The results demonstrated that the model also fit the data well (χ^2^ = 391.775, df = 242, χ^2^/df = 1.619, RMSEA = 0.059, CFI = 0.918, TLI = 0.903). The results also showed that moral courage moderated the effect of identification with supervisors on UPB (β = −0.149, *p* < 0.1, BC bootstrap 90% CI = [−0.282, −0.026], excluding 0) such that the positive relationship between identification with supervisors and UPB was weaker for employees with high rather than low moral courage. Figure 3 illustrated the moderating effect of moral courage on the relationship between identification with supervisors and UPB. Thus, H_3__a_ was supported in sample 2.

**FIGURE 4:**
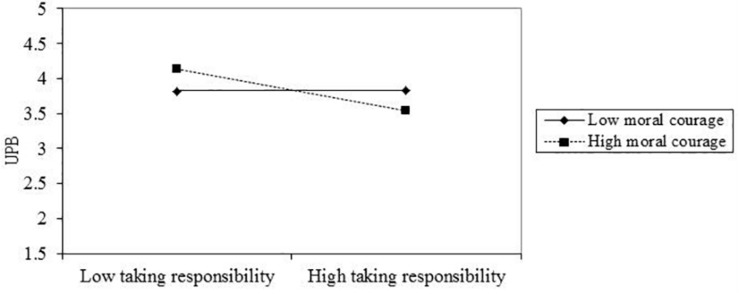
The hypothetical moderating effect of moral courage on the relationship between taking responsibility and UPB in Study 2.

The results also showed that moral courage moderated the relationship between taking responsibility and UPB (β = −0.186, *p* < 0.05, BC bootstrap 95% CI = [−0.338, −0.013], excluding 0) such that the negative relationship between taking responsibility and UPB was stronger for employees with high rather than low moral courage. Figure 4 illustrated the moderating effect of moral courage on the relationship between taking responsibility and UPB. Therefore, H_4__a_ was supported in sample 2.

##### Testing H_3__*b*_ and H_4__*b*_

The results showed that for the group with high and low moral courage, the indirect effect of moral leadership on UPB through identification with supervisors was significantly different (β = −0.080, *p* < 0.1, BC bootstrap 95% CI = [−0.183,−0.005], excluding 0). As shown in Table 9, when moral courage was low, the indirect effect of moral leadership on UPB through identification with supervisors was significant (indirect effect = 0.194, *p* < 0.01, BC bootstrap 95% CI = [0.094,0.350], excluding 0). However, when moral courage was high, the indirect effect of moral leadership on UPB through identification with supervisors was not significant (indirect effect = 0.058, *p* > 0.05, BC bootstrap 95% CI = [−0.051,0.216], including 0). The difference of the indirect effects between the group with low and high moral courage was significant (Δ indirect effect = 0.136, *p* < 0.1, BC bootstrap 95% CI = [0.008,0.311], excluding 0). Thus, H_3__b_ was supported.

**TABLE 9:** Summary of moderated mediation (Study 2).

**Moderator variable**	**Moral leadership (X) → identification with supervisors (M) →unethical pro-organizational behavior (Y)**		
	
	**First stage (P_MX_)**	**Second stage (P_YM_)**	**Indirect effects (P_MX_^∗^ P_YM_)**
Low moral courage (−1 SD)	0.562^∗∗∗^	0.344^∗∗∗^	0.194^∗∗^ (95% CI = [0.094,0.350])
High moral courage (+1 SD)	0.562^∗∗∗^	0.102	0.058 (95% CI = [−0.051,0.216])
Differences	0	0.242^†^	0.136^†^(95% CI = [0.008,0.311])

**Moderator variable**	**Moral leadership (X) → taking responsibility (M) →unethical pro-organizational behavior (Y)**		
	
	**First stage (P_MX_)**	**Second stage (P_YM_)**	**Indirect effects (P_MX_^∗^ P_YM_)**

Low moral courage (−1 SD)	0.119^∗∗^	–0.123	−0.015 (95% CI = [−0.079,0.023])
High moral courage (+ 1 SD)	0.119^∗∗^	–0.513^∗∗^	−0.061^∗^ (95% CI = [−0.157,−0.015])
Differences	0	0.390^∗^	0.046^†^(95% CI = [0.004,0.133])

**N* = 205, ^†^*p* < 0.1, ^∗^*p* < 0.05, ^∗∗^*p* < 0.01, ^∗∗∗^*p* < 0.001; bootstrap = 10000. Unstandardized path coefficients are reported. P_*MX*_, first-stage effect of predictor X on mediator (M); P_*YM*_, second-stage effect of M on Y. Tests of P_*MX*_, P_*YM*_, and P_*MX*_^∗^ P_*YM*_ are based on bias-corrected confidence intervals derived from bootstrap estimates.*

Then, for the group with high and low moral courage, the indirect effect of moral leadership on UPB through taking responsibility was also significantly different (β = −0.027, *p* > 0.05, BC bootstrap 95% CI = [−0.079,−0.002], excluding 0). As shown in Table 9, when moral courage was low, the indirect effect of moral leadership on UPB through taking responsibility was not significant (indirect effect = −0.015, *p* > 0.05, BC bootstrap 95% CI = [−0.079,0.023], including 0). When moral courage was high, the indirect effect of moral leadership on UPB through taking responsibility was significant (indirect effect = −0.061, *p* < 0.05, BC bootstrap 95% CI = [−0.157,−0.015], excluding 0). The difference of the indirect effects between the group with low and high moral courage was significant (Δ indirect effect = 0.046, *p* < 0.1, BC bootstrap 95% CI = [0.004,0.133], excluding 0). Thus, H_4__b_ was supported in sample 2.

Taken together, the above results provided support for our research model whether in the student sample or in the employee sample.

## General Discussion

### Summary of Main Findings

Drawing on Social Identity Theory, we developed and tested a model in how and when moral leadership affects UPB. We conducted two studies with two distinct samples based on a two-wave research design, thus increasing not only the external validity but also the internal validity of our research overall. The results demonstrate that: (1) Moral leadership affects UPB *via* two paths. One path is through identification with supervisors, revealing the promotion mechanism of an employee conducting UPB. Another path is through taking responsibility, revealing the suppression mechanism of an employee conducting UPB. (2) Moral courage, which moderates the mediating effects of identification with supervisors and taking responsibility upon the relationship between moral leadership and UPB, is a key boundary condition of moral leadership influencing UPB. Below, we discuss theoretical and practical implications, along with limitations and future directions of the present work.

### Theoretical Implications

Our study focuses on the antecedent, mediating mechanisms, and boundary condition of UPB, which offers the following theoretical contributions to literature on UPB, leadership and Social Identity Theory. First, the study expands the research on the antecedent of UPB. Most previous studies have examined the impacts of personal characteristics (e.g., Machiavellianism, psychological entitlement) (cf. Castille et al., 2018; Lee et al., 2019), workplace situational factors (e.g., job insecurity, social exclusion) (cf. Thau et al., 2015; Ghosh, 2017), and organizational factors (e.g., high performance work system, idiosyncratic deals) (cf. Ilie, 2012; Xu and Lv, 2018) on UPB, while the impact of leadership on UPB has not been sufficiently focused on (see Miao et al., 2013; Effelsberg et al., 2014; Kalshoven et al., 2016 as exceptions). Numerous organizational researchers have suggested that leadership is an important organizational context variable that shapes employees’ behavior (Chen et al., 2002; Tang and Naumann, 2015). Theoretically, UPB results from employees supporting or protecting their leaders and organizations, while moral leadership provides a passageway for employees identifying with and supporting leaders. Therefore, our study contributes to UPB literature through testing one of the important theoretical antecedents of UPB—moral leadership.

Second, our study brings a comprehensive understanding of moral leadership’s impact on UPB through an examination of its mechanisms and provides a deeper understanding of the double-edged sword effect of positive leadership on UPB from the lens of identification. Extant research focuses on the positive effect of moral leadership (Farh et al., 2006), while the negative side effect of moral leadership is largely unknown and unexplored. In order to gain a more comprehensive understanding of leadership’s realistic impact, Eisenbeiß and Boerner (2013) suggested that scholars should examine both the positive and the negative effects of leadership. As a response to the call from Eisenbeiß and Boerner (2013), we proposed both the positive and the negative paths and established the paradoxical effects of moral leadership influencing UPB, both of which provided a comprehensive understanding for the positive effect and possible risk of moral leadership. Moreover, previous studies discussed the effect of ethical leadership on UPB relying on the external guidance of ethical leadership (e.g., Miao et al., 2013), which was insufficient to explain the intrinsic motivation of employees conducting UPB. Meanwhile, two studies talked only about the dark side of positive leadership’s impact on UPB from the lens of social identification (e.g., Effelsberg et al., 2014; Kalshoven et al., 2016), which was insufficient to demonstrate a realistic picture of how positive leadership works. Prominent leadership theorists have called for a deeper study of how leadership style exerts effects on subordinates (Yukl, 2006). Lord and Brown (2004) have placed especial focus on the psychological effects on subordinates. With this in mind, our study is an initial attempt to understand the paradoxical effects of positive leadership on UPB from the lens of identification.

Third, although Social Identity Theory provides us an important perspective from which to examine the paradoxical effects of moral leadership, our study also expands Social Identity Theory by incorporating identification with supervisors and taking responsibility as mechanisms of moral leadership influencing UPB through which two different identities work together. Several studies have shown that positive leadership (e.g., ethical leadership, transformational leadership) is positively related to UPB through the shaping of subordinates’ social identities (c.f. Effelsberg et al., 2014; Kalshoven et al., 2016). However, an individual also has a personal identity that reflects his/her uniqueness (Brewer and Gardner, 1996). This study integrated two identification processes as two mechanisms stimulated by moral leadership. More precisely, moral leadership impacted UPB simultaneously through employees’ identification with supervisors and taking responsibility, respectively, as social identity reflecting employees’ belongingness and personal identity reflecting employees’ self-consistency.

Furthermore, when examining the effect of moral leadership on UPB through identification mechanisms, scholars should think about the role of moral courage. In this study, we theorize and empirically test that the positive relationship between moral leadership and UPB through identification with supervisors is weaker with employees of high rather than low moral courage, while the negative relationship between moral leadership and UPB through taking responsibility is stronger with employees of high rather than low moral courage. Thus, we may consider integrating moral courage into Social Identity Theory as a key boundary condition.

### Managerial Implications

Our findings also provide several practical implications. First, in the era of globalization, with increasingly frequent cultural communication between countries and regions, organizations have diverse values and they also confront the challenges from cultural conflict. Leaders in organizations should highlight the importance of ethical vision, mission, and values and create a culture with corporate social responsibility (May et al., 2015), pay more attention to setting a good example, and engage in role modeling for subordinates. Moreover, organizations should place emphasis on moral standards in leader selection in addition to other qualifications.

Second, there are a promotion mechanism and a suppression mechanism existing in the effect of moral leadership on UPB: identification with supervisors (the promotion mechanism) and taking responsibility (the suppression mechanism). We compared the mediation effect of identification with supervisors with that of taking responsibility and the results showed that the mediation role of identification with supervisors was stronger than that of taking responsibility. Therefore, moral leaders should not only set a good example for subordinates but also be aware of empowering subordinates to avoid blind allegiance and loyalty to the supervisors.

Third, researchers have called for a better understanding of what could promote ethical behavior in the workplace (Treviño et al., 2006). Our study demonstrated that moral courage could be an important psychological strength to eliminate blind identification with supervisors and enhance the effect of taking responsibility. Hence, organizations should not only recruit moral employees and provide training programs to boost employees’ moral courage (Hannah et al., 2011; Liao et al., 2018) but also create a good ethical environment to motivate employees’ moral courage and implement an ethical organizational culture to increase employees’ willingness to fight with immoral behavior (Yam et al., 2014).

### Limitations and Future Directions

There are also several limitations in our study and directions for future research. First, although we collected data in two phases, all variables were assessed by employees, which may result in CMB. We tested CMB with ULMC technique and found that CMB had no significant effects in our measurement. The results of a discriminant validity test also revealed the distinctiveness of the constructs. Future research should use longitudinal research design and collect data from various sources in order to reduce CMB and understand the real causal relationships between variables.

Second, we assessed two competing mediating mechanisms of moral leadership influencing UPB, which may not fully reflect the psychological mechanism of moral leadership’s impact on UPB. For example, Li et al. (2012) found that moral leadership is positively related to psychological empowerment, while psychological empowerment is positively related to UPB (Sun et al., 2018). Hence, we predict that psychological empowerment is likely to be a mediator of moral leadership’s impact on UPB. Future research can explore other possible mediators based on different theoretical perspectives.

Third, we investigated two samples in China. However, moral leadership exists widely in regions with Confucian culture. Future research can empirically test our research model using samples from other countries (e.g., Japan) and other regions (e.g., some areas of the Pacific Islands) with Confucian culture to improve the ecological validity of our study.

Fourth, although UPB is financially advantageous to the organization in the short term, it harms the organization in the long run (Umphress and Bingham, 2011). When employees judge what a “beneficial” action to the organization is, they may depend on what time horizon they are using in their assessment. Therefore, we could consider the effect of window of time in future study.

## Conclusion

In closing, using the field method, we found empirical evidence that moral leadership promotes UPB through increasing identification with supervisors while also reducing UPB *via* increasing taking responsibility. We also found that the mediating effect of identification with supervisors upon the relationship between moral leadership and UPB is weaker for employees with high rather than low moral courage, while the mediating effect of taking responsibility upon the relationship between moral leadership and UPB is stronger for employees with high rather than low moral courage. Our results extend knowledge of moral leadership and UPB through the lens of identification. Considering the importance of moral leadership’s effect on UPB, managers should not only possess personal virtues but also set a good example for subordinates to follow, which helps the organizations develop in a healthy and ordered manner.

## Data Availability Statement

The raw data supporting the conclusions of this study will be made available to any qualified researcher, without undue reservation.

## Ethics Statement

YW collected all the data in the study. The procedure of data collection has been described in detail in the study. The study was carried out in accordance with the ethical standards of the Research Ethics Review Committee at the Business School of Beijing Normal University and with the Declaration of Helsinki. The study was approved by the Beijing Social Science Foundation and funded as an important research program. In addition, the protocol was approved by the Research Ethics Review Committee at the Business School of Beijing Normal University. Moreover, we received permission from the university principal and company leader prior to recruiting participants. All participants read and gave written informed consent document before participating in the study. All participation was voluntary. To protect all participants, all responses were anonymous.

## Author Contributions

YW and HL participated in the design of this study. YW performed the statistical analysis and drafted the manuscript. HL provided comments on different versions of the manuscript. Both authors read and approved the final manuscript.

## Conflict of Interest

The authors declare that the research was conducted in the absence of any commercial or financial relationships that could be construed as a potential conflict of interest.

## APPENDIX

### Measurement Items

**Taking Responsibility**

(1) It is my obligation to be responsible for the scope of my duties. (0.728)
(2) I would be responsible for my mistake. (0.741)
(3) I would not defer responsibility to others when I did something wrong. (0.720)

**Moral Leadership** (Cheng et al., 2000)

(1) He is honest, and doesn’t conduct the self-dealing behavior.
(2) He is impartial to everyone.
(3) He doesn’t pull strings for personal gain.
(4) He sets an example for others.
(5) He can lead by example.

**Identification with Supervisors (Shamir et al., 1998)**

(1) I have complete faith in him.
(2) I respect him.
(3) I am proud to be under his command.
(4) I trust his judgment and decisions completely.
(5) He represents values that are important to me.
(6) My values are similar to his values.
(7) He is a model for me to follow.

**Moral Courage (Hannah et al., 2011)**

(1) I confront my peers if they commit an unethical act.
(2) I confront a leader if she/she commits an unethical act.
(3) I always state my views about ethical issues to my leaders.
(4) I go against the group’s decision whenever it violates my ethical standards.

**Unethical Pro-organizational Behavior (Umphress et al., 2010)**

(1) If it would help my organization, I would misrepresent the truth to make my organization look good.
(2) If it would help my organization, I would exaggerate the truth about my company’s products or services to customers and clients.
(3) If it would benefit my organization, I would withhold negative information about my company or its products from customers and clients.
(4) If my organization needed me to, I would give a good recommendation on the behalf of an incompetent employee in the hope that the person will become another organization’s problem instead of my own.
(5) If my organization needed me to, I would withhold issuing a refund to a customer or client accidentally overcharged.
(6) If needed, I would conceal information from the public that could be damaging to my organization.
